# New Insights on Drought Stress Response by Global Investigation of Gene Expression Changes in Sheepgrass *(Leymus chinensis*)

**DOI:** 10.3389/fpls.2016.00954

**Published:** 2016-06-30

**Authors:** Pincang Zhao, Panpan Liu, Guangxiao Yuan, Junting Jia, Xiaoxia Li, Dongmei Qi, Shuangyan Chen, Tian Ma, Gongshe Liu, Liqin Cheng

**Affiliations:** ^1^Key Laboratory of Plant Resources, Institute of Botany, The Chinese Academy of SciencesBeijing, China; ^2^College of Life Sciences, University of Chinese Academy of SciencesBeijing, China

**Keywords:** sheepgrass, drought stress, RNA-seq, ABA-dependent pathway, transcription factors

## Abstract

Water is a critical environmental factor that restricts the geographic distribution of plants. Sheepgrass [*Leymus chinensis*, (Trin.) Tzvel] is an important forage grass in the Eurasia Steppe and a close germplasm for wheat and barley. This native grass adapts well to adverse environments such as cold, salinity, alkalinity and drought, and it can survive when the soil moisture may be less than 6% in dry seasons. However, little is known about how sheepgrass tolerates water stress at the molecular level. Here, drought stress experiment and RNA-sequencing (RNA-seq) was performed in three pools of RNA samples (control, drought stress, and rewatering). We found that sheepgrass seedlings could still survive when the soil water content (SWC) was reduced to 14.09%. Differentially expressed genes (DEGs) analysis showed that 7320 genes exhibited significant responses to drought stress. Of these DEGs, 2671 presented opposite expression trends before and after rewatering. Furthermore, ~680 putative sheepgrass-specific water responsive genes were revealed that can be studied deeply. Gene ontology (GO) annotation revealed that stress-associated genes were activated extensively by drought treatment. Interestingly, cold stress-related genes were up-regulated greatly after drought stress. The DEGs of MAPK and calcium signal pathways, plant hormone ABA, jasmonate, ethylene, brassinosteroid signal pathways, cold response CBF pathway participated coordinatively in sheepgrass drought stress response. In addition, we identified 288 putative transcription factors (TFs) involved in drought response, among them, the WRKY, NAC, AP2/ERF, bHLH, bZIP, and MYB families were enriched, and might play crucial and significant roles in drought stress response of sheepgrass. Our research provided new and valuable information for understanding the mechanism of drought tolerance in sheepgrass. Moreover, the identification of genes involved in drought response can facilitate the genetic improvement of crops by molecular breeding.

## Introduction

Drought has induced the reduction of global terrestrial net primary production (NPP) in the past several years, with an important impact on food security (Zhao and Running, 2010, 2011; Medlyn, 2011; Samanta et al., 2011). Food demand for the increasing population further aggravates the effects of drought. Water deficit will become more serious with increasing temperature in the future, posing a greater threat to crop yields (Harrison et al., 2014). The development of crops for enhanced drought resistance is a promising approach to alleviate this crisis (Farooq et al., 2009). Luckily, plants display amazing diversity, and some of them are able to adapt well to severe environments. Therefore, selecting plant materials with natural drought tolerance and uncovering their mechanisms of resistance to stress are likely to pave the way for designing crop plants with drought resistance.

The influence of drought on growth, nutrient metabolism, photosynthesis, and crop yields was reviewed by Farooq et al. (Farooq et al., 2009). To cope with drought stress, tolerant plants initiate responsive mechanisms at different levels including the whole plant as well as physiological and molecular levels. The physiological responses of plants to drought stress mainly include the production of osmotic protective compounds, antioxidants and growth regulators (Zhu, 2002; Yamada et al., 2005a; Fazeli et al., 2007; Farooq et al., 2008; Karatas et al., 2014; Fahad et al., 2015). Abscisic acid (ABA), an important growth regulator, is the central signal molecule that responds to abiotic stresses and coordinates a complex gene regulatory network (Yamaguchi-Shinozaki and Shinozaki, 2006; Kim et al., 2010). ABA-dependent and ABA-independent pathways have been proposed in past studies (Yamaguchi-Shinozaki and Shinozaki, 2006; Ahuja et al., 2010; Raghavendra et al., 2010). Of these pathways, TFs play crucial roles in the adaptation of plants to environmental stresses by dominating the activity of multiple stress-responsive genes that exhibit attractive targets for application in molecular breeding (Seki et al., 2003; Yamaguchi-Shinozaki and Shinozaki, 2006; Morran et al., 2011). The overexpression of *DREB1A* improved the drought stress tolerance of rice and wheat (Pellegrineschi et al., 2004; Oh et al., 2005). Because of their importance, TFs have been isolated in many plant species such as rice (Wu et al., 2005; Wang et al., 2012; Hirano et al., 2013), wheat (Cai et al., 2011; Okay et al., 2014; Chen et al., 2015), maize (Zhang et al., 2008; Wang G. F. et al., 2010; Fountain et al., 2012), and barley (Xue, 2003; Tombuloglu et al., 2013; Matsumoto et al., 2014). In addition, aquaporins and dehydrins have been reported to be involved in drought tolerance in numerous species (Hu et al., 2010; Shatil-Cohen et al., 2011; Grondin et al., 2016; Perdiguero et al., 2015).

RNA-seq is a recently developed approach to transcriptome profiling that has many advantages, including high throughput, suitability for species of unknown genomic sequence, and low background signal (Wang et al., 2009). At the same time, several analytical software programs have been developed, such as Tophat (Trapnell et al., 2009), RobiNA (Lohse et al., 2012), and DEGseq (Wang L. K. et al., 2010). Therefore, it has already been applied to many species, such as *Arabidopsis thaliana* (Loraine et al., 2013), *Oryza sativa* (Xu H. et al., 2012), *Zea mays* (Kakumanu et al., 2012), *Saccharomyces cerevisiae* (Nagalakshmi et al., 2008), and others.

Sheepgrass is a perennial forage grass of the Poaceae family that is distributed widely in Eurasia and adapts well to drought, cold, saline and alkaline conditions (Chen et al., 2013; Zhai et al., 2014). It has been reported that this species could survive when the soil moisture was <6% in dry seasons (Wang et al., 2008). Phylogenetic analysis showed that sheepgrass had close phylogenetic relationships with wheat and barley (Chen et al., 2013). Sheepgrass is an allotetraploid (2*n* = 4x = 28) grass, which has a very large genome (9.65 Gb for a haploid genome), whole-genome sequencing is not complete for sheepgrass until now. To better understand the molecular mechanism of stress tolerance in sheepgrass, a series of transcriptome analyses were conducted over recent years (Chen et al., 2013, 2014; Sun et al., 2013; Huang et al., 2014; Zhai et al., 2014). Many valuable genetic resources were identified based on these transcriptomic data. To further understand how these genes function in plants, a number of stress-responsive genes have been characterized. Of these genes, *LcDREB3a* improved the drought and salt stress tolerance of transgenic *Arabidopsis* (Peng et al., 2011); *LcMYB1, LcDREB2, LcSAIN1*, and *LcSAIN2* improved the salt stress tolerance of transgenic lines (Cheng et al., 2013; Peng et al., 2013; Li et al., 2013a,b); and *LcFIN1*, regulated by LcCBF1, enhanced tolerance to low temperature stress in transgenic plants (Gao et al., 2016). All of these genes are promising candidates for improving crop stress tolerance in future work. However, the effects of drought stress on global changes in gene expression have not yet been revealed in sheepgrass.

In this study, pot culture experiments were conducted in a greenhouse with 2-month old seedlings. Sheepgrass still survived when the SWC was reduced to 14.09%, which has a significant effect on the growth and biomass of the aboveground part. To understand the mechanism of drought tolerance and identify candidate genes that could potentially be used to enhance crop drought resistance, RNA-seq analysis was performed. We analyzed the changes in gene expression in three moisture states. Compared with the control group, 7320 DEGs were identified as drought-responsive genes, and 2671 presented an opposite expression trend after rewatering. Furthermore, ~680 putative sheepgrass-specific water responsive genes were revealed. A total of 288 putative TFs were identified among the DEGs as potentially related to drought response. A comprehensive analysis of function and expression showed that the genes of the “ethylene, abscisic acid (ABA), Jasmonates (JAs) and Brassinosteroids (BRs) mediated signaling pathway” were activated at different levels; however, photosynthesis-related genes were greatly down-regulated. Moreover, the genes involved in the responses to oxidative stress and osmotic stress, including cold, salt, wounding, water and water deprivation, were intensively up-regulated by drought stress.

## Methods

### Plant materials and growth conditions

We used vermiculite and nutrition soil (2:1) to cultivate the seedlings. Two-month-old seedlings of sheepgrass were cut back to ~2 cm height above the soil level and supplied with fertilizer and sufficient water. After 15 days' growth, they were used for a pot culture experiment in the greenhouse. During the experimental periods of experiment, the control groups were watered adequately, while the experimental groups were treated with natural drought stress for 28 days after the last saturation with water. At the point when most leaves were wilted, the water supply was restored for 15 days. The heights of seedlings, soil water content, and fresh weight, dry weight of the aboveground part and the aboveground part's water content (AWC) were measured from the fourteenth (14th) day. Three biological repeats were measured for each sample. Based on the lowest AWC and a relevant morphological trait, a quick drought stress experiment was performed using 1-month-old seedlings. Three samples of sheepgrass leaves were collected in different conditions (control, drought, rewatering) for RNA-seq analysis and qRT-PCR.

### Soil water content determinations

Each soil sample taken was weighed immediately to obtain the wet weight (W1). Then, after drying the samples at 105°C for 24 h to standing weight (W2), the SWC was calculated according to the following formula:

(1) $$\begin{array}{l} \text{SWC}=\frac{W1-W2}{W1}\times 100\% \end{array}$$

### Aboveground part water content determinations

We excised and weighed the aboveground part immediately to obtain the fresh weight (FW). After oven drying at 105°C for 24 h, the dry weight (DW) was taken, and AWC was calculated according to the following formula:

(2) $$\begin{array}{l} \text{AWC}=\frac{FW-DW}{FW}\times 100\% \end{array}$$

### RNA extraction and cDNA synthesis

Total RNA was extracted from the leaves using Trizol reagent (Invitrogen, Carlsbad, CA, USA) according to the manufacturer's instructions. The quality of the total RNA was determined using a 2100 Bioanalyzer (Agilent Technologies, Santa Clara, CA, USA) and 1% agarose gel electrophoresis. The acceptable samples (30 μg) were digested with DNase I (Takara, Japan) at 37°C for 30 minute. The mRNAs were isolated from the total RNA using DynabeadsOligo (dT) 25 (Life, America), and the cDNA library was constructed with 100 ng purified mRNA employing the NEBNextUltraTM RNA Library Prep Kit for Illumina (NEB, America) according to the manufacturer's instructions. Bidirectional sequencing was applied using an IlluminaHiseq™2500.

### qRT-PCR

Twelve contigs with differential expression were selected to confirm the RNA-seq results by qRT-PCR. The primers are listed in Table S10. The qRT-PCR of each contig was performed in triplicate according to the SYBR PremixExTaq™ protocol (TaKaRa) on a LightCycler480 Real-Time PCR System (Roche). The qRT-PCR results were analyzed by the system software. To compare qRT-PCR data with RNA-seq results, the log2 fold-change in expression of the drought-stressed samples was calculated relative to the control samples.

### Acquisition of clean reads and mapping

The low-quality reads (about 1.5–1.8%) and adaptors were filtered out using the program FASTX-Toolkit (http://hannonlab.cshl.edu/fastx_toolkit/), and sequence quality was evaluated using Fastqc (http://www.bioinformatics.babraham.ac.uk/projects/fastqc/). The clean reads were mapped to the reference transcriptome dataset (NCBI Sequence Read Archive SRA065691) obtained by Roche 454 pyrosequencing technology (Chen et al., 2013) using bowtie. The total mapped reads were used for further analysis.

### Comparative analysis between control and treatment samples

For comparative analysis of the expression between two groups, the clean reads were used to mapping to the transcriptome assembly with BOWTIE program according to the default parameters. The numbers of reads that matched to the unique gene from two samples were calculated separately and subsequently transformed into RPKM (Reads Per Kilobases per Million reads). Significant differences in gene expression between the two groups were determined using the package DEGseq (Wang L. K. et al., 2010) in the statistical environment R (version 3.20) with the MA-plot-based method and Random Sampling model (MARS; Wang L. K. et al., 2010). The raw p values were corrected for multiple tests using the False Discovery Rate (FDR), according to the method of Benjamini and Hochberg (1995). Genes with an “FDR<0.001” and a “fold change >2” were deemed to be significantly differentially expressed between the two samples. The RPKM values of related genes were used to plot heatmaps.

### Functional annotation and classification

To predict the possible functions and biological pathways of the genes, we annotated the genes using the following databases: the NR protein database (NCBI) and SwissProt database, the Gene Ontology (GO), the Kyoto Encyclopedia of Genes and Genomes (KEGG) and the Clusters of Orthologous Groups database (COG). The specific methods of these annotations were as described in Zhou et al. (2014). Pathways and GO function enrichment analyses were conducted based on a hypergeometric distribution (Sun et al., 2013). To investigate the roles of transcription factors (TFs) in the response to drought stress, we downloaded the transcription factors of *Oryza sativa subsp. japonica* and *Arabidopsis thaliana* from the Plant Transcription Factor Database (http://planttfdb.cbi.pku.edu.cn/) to construct a local database. Then, we searched the database with the protein sequences of DEGs by blastp at the threshold 1e-10. The results of blastp were extracted and counted using Perl scripts.

## Results

### Test of sheepgrass tolerance to drought stress

**Figure 1:**
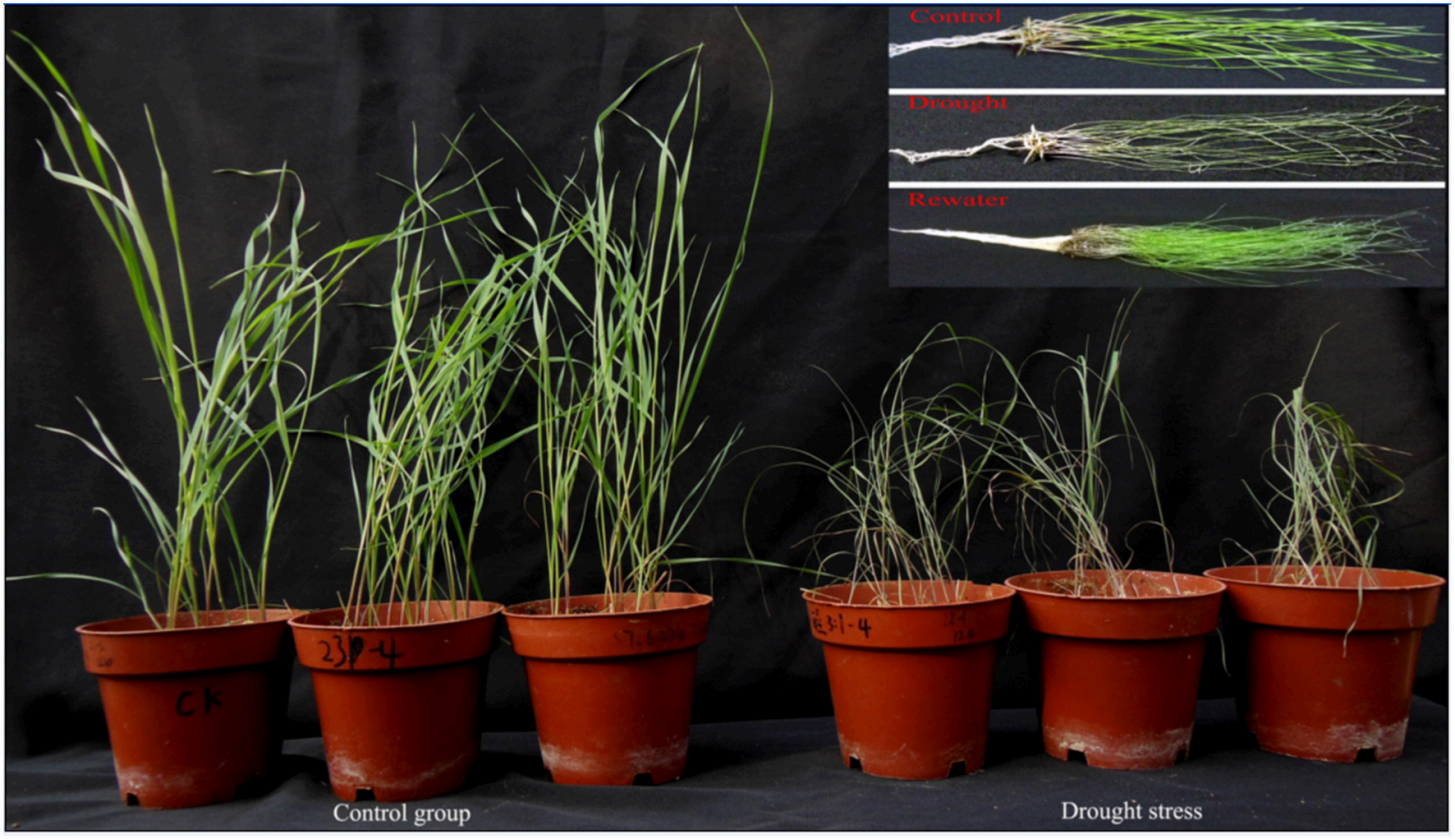
**Photos of sheepgrass under different growth conditions**. The control groups were watered adequately, drought stress groups were treated withdraw water in the pot. The control groups and drought stress groups seedlings were measured the heights of seedlings, soil water content, and fresh weight and dry weight of the aboveground part. The one-month-old seedlings of control, drought and rewater conditions were used for RNA-seq and further qRT-PCR validation.

**Figure 2:**
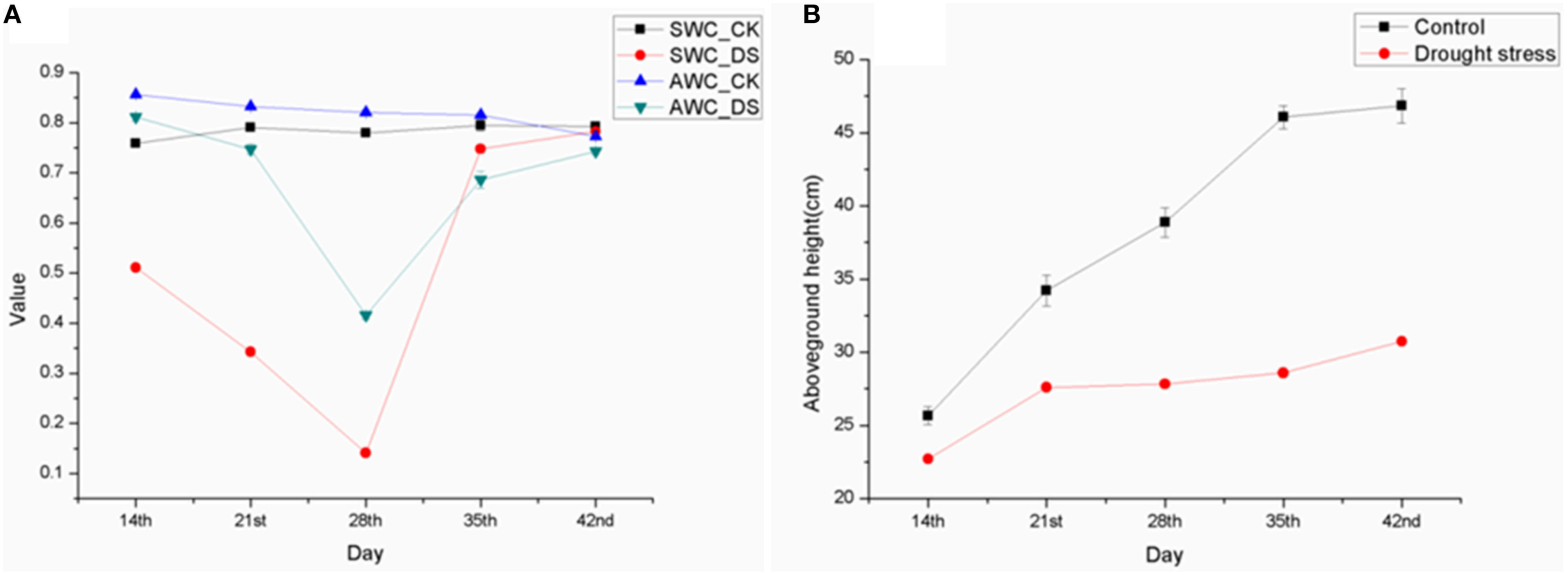
**Soil water content, aboveground part water content and height**. **(A)** Soil water content and aboveground part water content. Abscissa indicates the day of collecting materials; ordinate indicates the value of water content (0.5 namely 50%); SWC means Soil Water Content; AWC means Aboveground-part Water Content; **(B)** Aboveground part height of in control and drought stress. DS means Drought Stress treatment; CK means Control check. Data are obtained from three independent repeats and are expressed as the means ± SE.

Sheepgrass seedlings still survived under drought stress on the 28th day when the SWC was reduced to 14.09%, although most of the leaves wilted and almost stopped growing (Figures 1, 2). Interestingly, most of these grasses were able to turn green and recover growth after rewatering. The changes in SWC had a great influence on plant growth and biomass (dry weight). The rate of growth (slope of curve) declined dramatically from the 21st to the 28th day as the SWC decreased from 34.29 to 14.09% (Figure 2). The specific data on fresh weight, dry weight and aboveground height changes are shown in Table S1.

### Statistics and evaluation of original data

In this study, RNA-seq was performed to investigate the transcript abundance changes in sheepgrass subjected to dehydration and subsequent rewatering, total mapped reads were used for Differential Gene Expression analysis, and ~17.8 million clean reads accounting for over 98% of raw reads were obtained after removing low-quality reads (about 1.5–1.8%) and adaptors, information of the yield and quality per sample was in Table 1, Table S2 and Additional image 2. The sequencing depth was saturated when the number of reads for each sample reached ~5–6 million, as few new genes were detected, and the number of detected genes reached a plateau (Additional image 1), and sequence data were deposited in the NCBI Sequence Read Archive (SRA340247). Next, the clean reads were mapped to the reference transcriptome dataset (NCBI SRA065691) generated by a Roche 454 GS FLX sequencer using the Newbler 2.5 (pl) assembly program, and transcriptome reference contained a total of 87,214 unigenes, including 32,416 contigs (=100 bp) and 54,798 singletons (= 300 bp), the mean contig size and N50 were 607 and 813 bp, respectively (Chen et al., 2013). The total mapped reads were used to estimate the expression levels of genes, the details of alignment metrics were in Table 1.

**Table 1:** **Statistic of original data**.

**Sample**	**L1**	**L2**	**L3**
Raw Reads (pair)	6271830	6292631	5576770
Clean reads(pair)	6180085	6177255	5483463
Average length(bp)	2^*^100	2^*^100	2^*^100
Raw data	1.25G	1.26G	1.12G
Clean data	1.24G	1.24G	1.10G
Read 1 Q20	97.12%	97.29%	97.33%
Read 2 Q20	93.36%	94.49%	94.81%

*L1, leaf control; L2, leaf drought; L3, leaf rewater*.

### Identification of genes responding to drought stress

**Figure 3:**
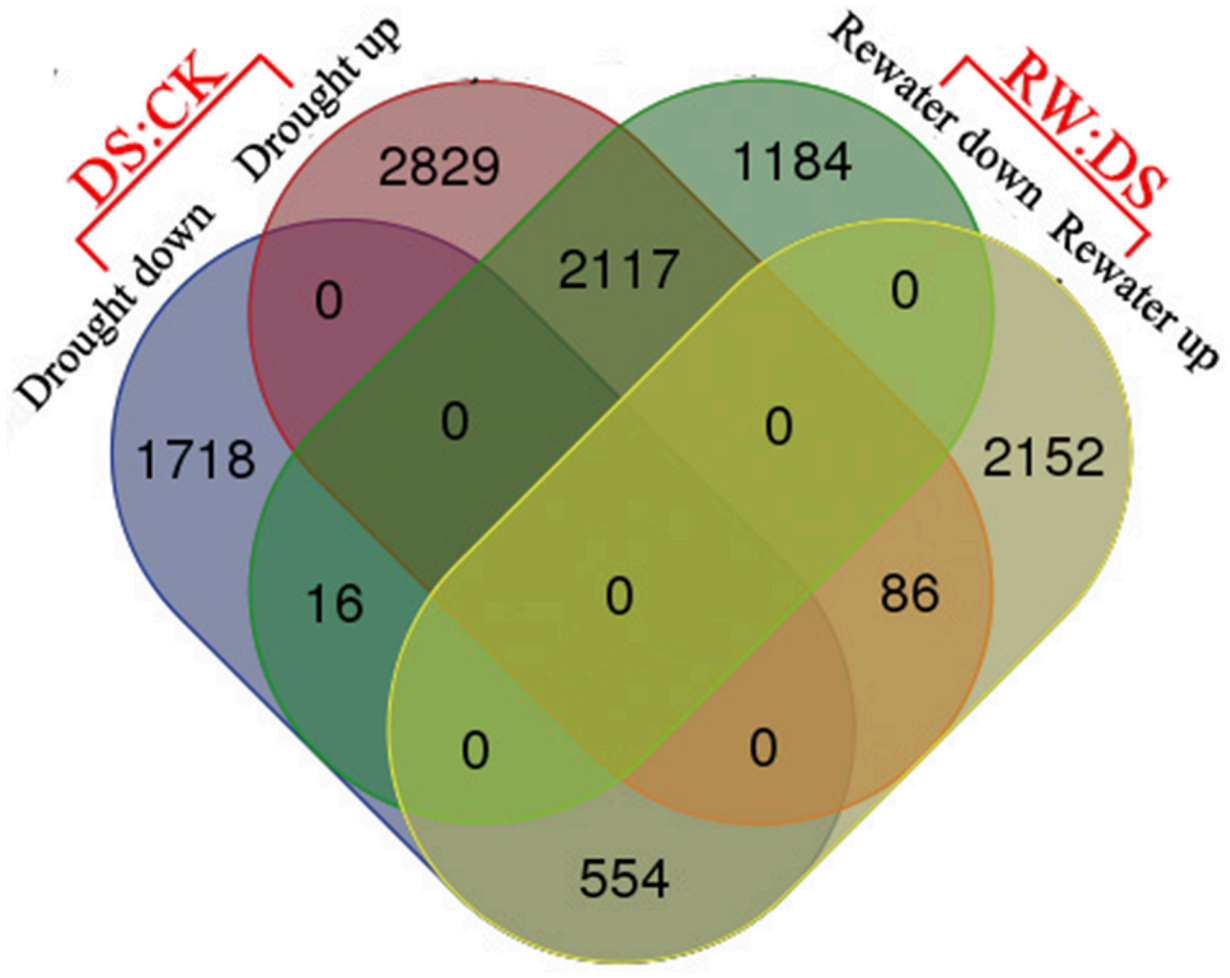
**Venn diagram analysis of differentially expressed genes**. Numbers of genes expressed differentially are shown in the diagram; DS means Drought Stress treatment; CK means Control check; and RW means Rewatering.

The expression abundances of each gene appearing in two libraries were used to determine the expression changes of the genes in response to drought stress. We discovered 7320 differentially expressed genes (DEGs) in leaves in response to drought. Compared to the control group, 5032 (68.74%) genes were induced and 2288 (31.26%) genes were repressed by drought. Among the 5032 induced genes, 2117 exhibited down-regulation after rewatering, while 554 of the 2288 repressed genes were up-regulated after rewatering (Figure 3, Tables S3–S5).These 2117 and 554 genes may play important roles in response to plant water content changes. After filtering, we found that many genes specifically expressed by more than 2^4^-fold were predicted to be related to plant stress tolerance and photosynthesis (Table 2), furthermore, ~680 genes (of fold-change > 16) that had no functional annotation were putative Sheepgrass-specific water responsive genes (Table S14). To further reveal the functions of these DEGs, function classification was performed based on multiple databases.

**Table 2:** **Partial of the specific expressed more than 2^**5**^-fold genes related with the plant drought stress response**.

**Gene**	**Length**	**Function**	**Log2 (drought/control)**	***q*-Value**
contig07984	746	9-cis-epoxycarotenoid dioxygenase(*Aegilops tauschii*)	7.019604	2.03E-30
2-GH8N3EB02F4DVO	393	9-cis-epoxycarotenoid dioxygenase 2 (*Hordeum vulgare* subsp. Vulgare)	6.643228	2.15E-12
contig41467	1905	Beta-glucosidase 31 (*Triticum urartu*)	5.227451	0
contig08894	1094	Beta-glucosidase 6 (*Aegilops tauschii*)	5.799387	1.44E-240
contig38471	1234	Trehalose-phosphate phosphatase (*Triticum urartu*)	5.994451	2.73E-46
contig33109	1038	Delta-1-pyrroline-5-carboxylate synthase(P5CS) (*Triticum urartu*)	6.132613	4.66E-34
contig60066	689	Delta-1-pyrroline-5-carboxylate synthase (P5CS)(*Triticum urartu*)	7.08234	1.29E-31
contig15454	1943	ACC synthase (*Hordeum vulgare*)	9.246569	1.61E-56
contig19955	3333	Serine/threonine-protein kinase CTR1 (*Aegilops tauschii*)	5.469831	2.74E-286
contig42160	1009	Serine/threonine-protein kinase CTR1 (*Aegilops tauschii*)	5.972536	1.31E-90
contig59644	419	Serine/threonine-protein kinase CTR1 (*Triticum urartu*)	5.861652	1.93E-28
contig86539	1307	Ethylene responsive transcription factor 6 (*Triticum durum*)	7.710973	2.75E-46
contig50565	1010	Ethylene responsive transcription factor 6 (*Triticum durum*)	9.14736	1.77E-53
contig19651	1776	12-oxophytodienoate reductase 2 (*Aegilops tauschii*)	5.99652	3.06E-214
contig37424	1301	Jasmonate-induced protein (*Triticum aestivum*)	5.362515	0
contig11922	1400	Jasmonate-induced protein (*Triticum aestivum*)	5.447086	0
contig40232	789	Dehydrin DHN3 (*Aegilops tauschii*)	7.453904	0
contig38960	849	Dehydrin 7 (*Hordeum vulgare* subsp. Vulgare)	7.129315	3.20E-192
contig46601	473	Drought acclimation dehydrin WZY2 (*Agropyron cristatum*)	6.710973	4.20E-25
contig06164	817	Drought acclimation dehydrin WZY2 (Agropyron cristatum)	6.410394	7.73E-41
contig35001	718	Dehydrin WZY1-2 (*Triticum aestivum*)	5.331548	0
contig90047	1452	Dehydrin WZY1-2 (*Triticum aestivum*)	5.471026	0
contig36997	801	Dehydrin WZY1-2 (*Triticum aestivum*)	5.514573	0
contig37235	1163	Dehydrin WZY1-2 (*Triticum aestivum*)	5.648766	0
contig02998	914	Late embryogenesis abundant proteinLea14-A (*Triticum urartu*)	8.398116	2.39E-69
2-GH8N3EB02J6KME	486	LEA protein (*Bromus inermis*)	7.80077	9.38E-25
contig45369	596	Early salt stress and cold acclimation-induced protein 2-1 (*Lophopyrum elongatum*)	5.639756	1.99E-191
contig43640	568	CBFII-5.3 (*Triticum aestivum*)	8.291517	6.46E-33
contig44268	1049	Cold acclimation protein WCOR410 (*Agropyron cristatum*)	5.460596	0
contig37840	778	Cold regulated protein (*Triticum aestivum*)	6.826305	0
contig87121	887	Cold regulated protein (*Triticum aestivum*)	10.45947	0
contig42056	1033	COLD shock protein CS66 (*Triticum urartu*)	11.18119	1.54E-160
contig37667	557	cold-regulated gene cor39	6.223286	0
contig42750	531	Peroxidase 2 (*Triticum urartu*)	8.255671	3.05E-32
contig60678	259	Peroxidase 2 (*Triticum urartu*)	7.410394	1.27E-38
contig15835	1047	Peroxidase 3 (*Aegilops tauschii*)	5.326493	6.68E-77
contig44866	934	Peroxidase 56 (*Triticum urartu*)	5.35557	2.61E-49
contig41960	919	Thioredoxin peroxidase (*Triticum aestivum*)	9.737204	4.27E-74
3-GH8N3EB01DSR8V	471	Polyamine oxidase (*Aegilops tauschii*)	−5.020686	7.47E-74
contig92996	260	Rubisco large subunit, partial (chloroplast) (*Paederia pertomentosa*)	−11.18521	1.22E-154
contig92997	203	Rubisco large subunit (*Convolvulus sabatius* subsp. mauritanicus)	−10.85487	5.78E-130
contig85991	268	Rubisco activase B, chloroplastic (*Triticum urartu*)	−6.705184	1.15E-164
contig90018	1577	Sugar transport protein 13 (*Aegilops tauschii*)	5.913809	3.30E-159
4-GJVU7SP04ITTBI	287	Beta-amylase 1, chloroplastic (*Triticum urartu*)	8.255671	3.05E-32
contig92993	2742	acetyl-CoA carboxylase beta subunit (*Corynocarpus laevigata*)	−12.51459	0

### GO annotation

**Figure 4:**
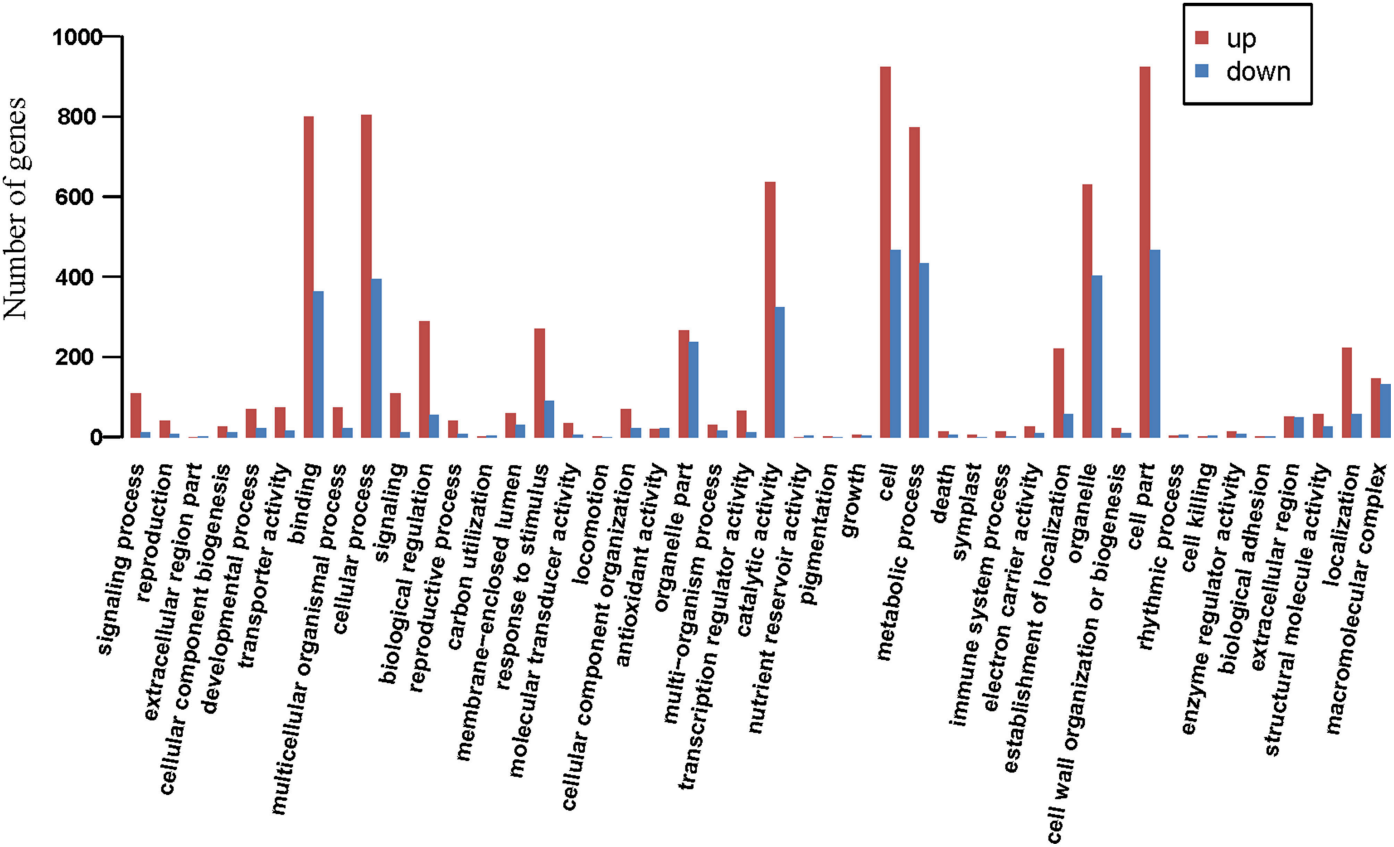
**GO functional annotation**. Abscissa indicates the functional categories; ordinate indicates the number of genes.

Of 7320 DEGs, a total of 1770 could be assigned GO identifiers classified into the three categories of “biological processes,” “molecular functions,” and “cellular components”. In many cases, a contig was involved in multiple functions. Among these functions, “metabolic process,” “cellular process,” “binding function,” “catalytic function,” “cell” and “cell part” were active in responding to drought (Figure 4). Further analysis showed that genes of “ABA biosynthetic process,” “ABA mediated signaling pathway,” “ethylene biosynthetic process,” “ethylene mediated signaling pathway,” “jasmonic acid (JA) biosynthetic process,” “JA mediated signaling pathway,” “proline biosynthetic process,” and “response to osmotic stress/oxidative stress/water/water deprivation/wounding” were strongly induced. At the same time, genes involved in the molecular functions of “transcription factor activity” and “unfolded protein binding” were up-regulated to varying degrees. However, among the cellular components, all genes related to “photosystem I/II,” “photosystem I/II reaction center,” “photosystem II antenna complex,” and “PSII associated light harvesting complex II” and most genes related to “chloroplast thylakoid” were strongly repressed by drought. In addition, genes of the “reductive pentose phosphate cycle” were down-regulated greatly, while genes of “carbohydrate transport,” “carbohydrate phosphorylation,” and “sucrose/galactose transport,” were up-regulated (Table S6). These changes provided a molecular basis for understanding the character of sheepgrass tolerance to drought and why photosynthesis declines and net biomass decreases under severe drought conditions.

### Pathway analysis of DEGs

**Figure 5:**
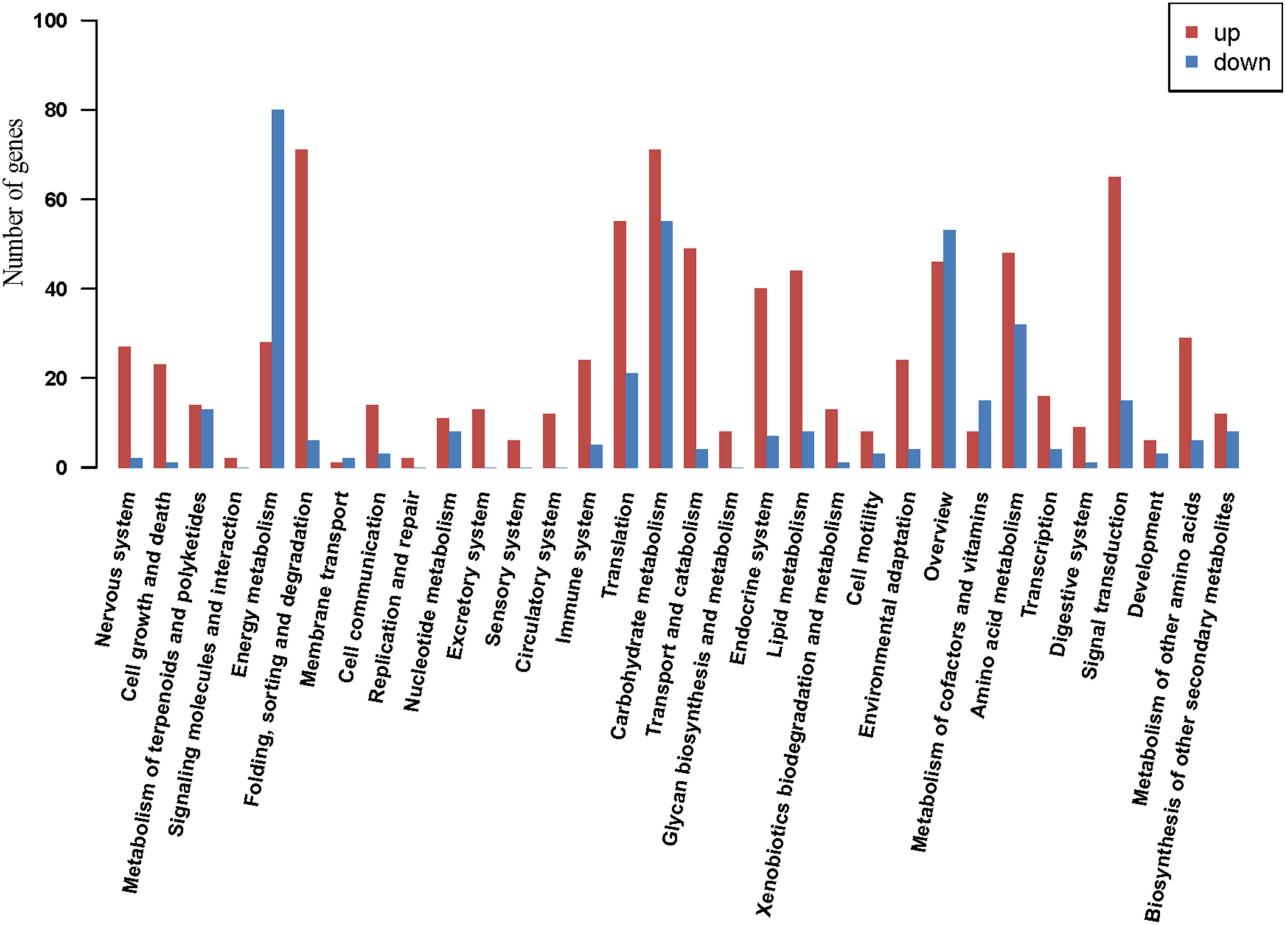
**KEGG pathway analysis**. Abscissa indicates the KEGG pathway; ordinate indicates the number of genes assigned to a specific pathway.

**Figure 6:**
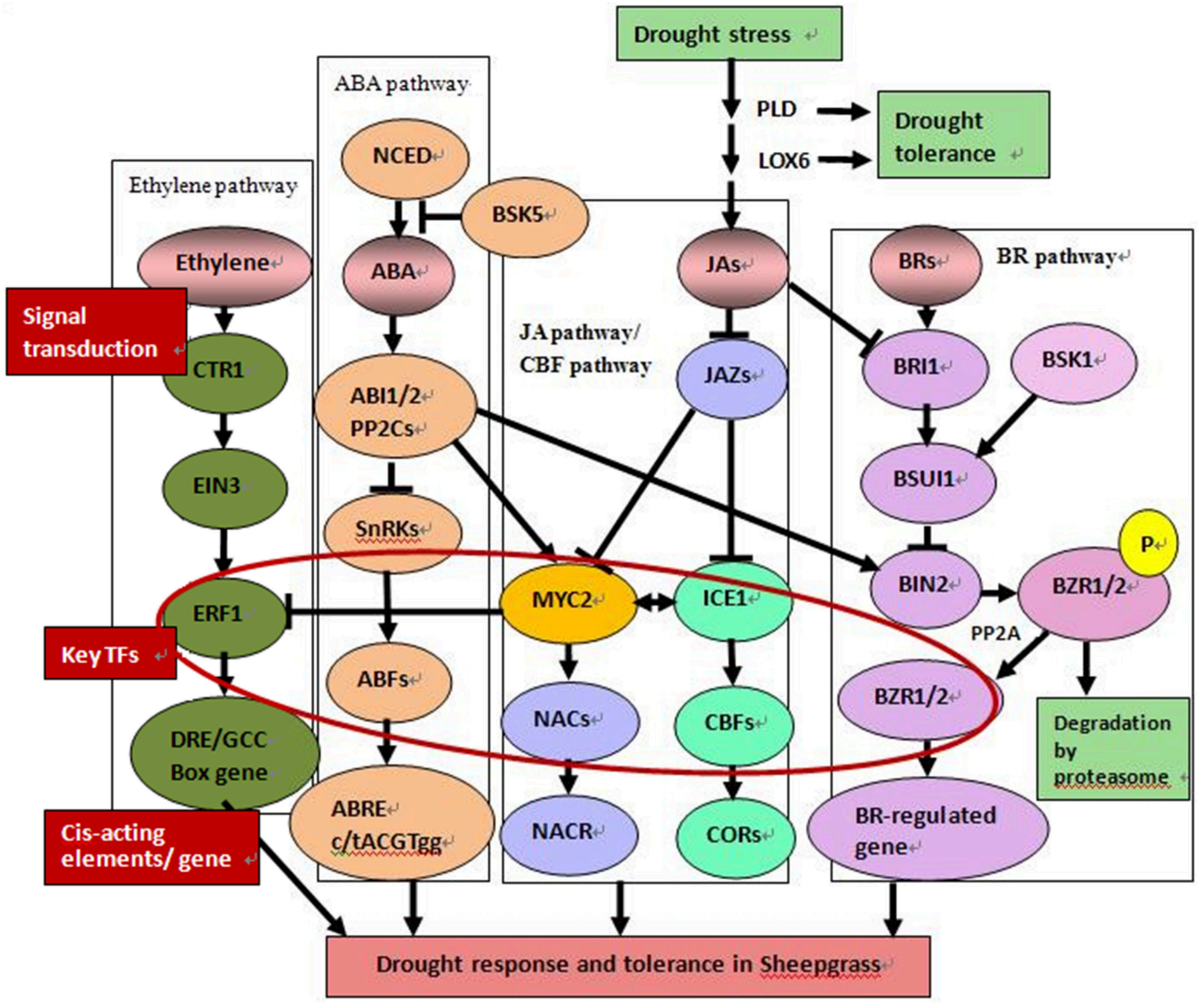
**Schematic diagram of potential drought response signaling pathways in sheepgrass**. (A) ABA pathway. NCED, nine-cis-epoxycarotenoid dioxygenase; PP2C, protein phosphatase 2C; SnRK2s, sucrose non-fermenting 1-related protein kinase 2s; ABF, ABA-responsive elements-binding factor; ABRE, ABA-responsive element-binding protein. (B) CBFs pathway. ICE1, inducer of CBF expression 1; CBF, C-repeat binding factor; COR, cold responsive genes. (C) Jasmonate pathway. LOX, linoleate 13S-lipoxygenase; PLD, phospholipase D; JAZ, jasmonate-zim-domain protein 11. (D) Ethylene pathway. EIN3, ethylene-insensitive protein 3; ERF, ethylene-responsive factor-like transcription factor. (E) Brassinosteroid pathway. BRI1, Brassinosteroid insensitive 1; BSU1, BRI1-suppressor 1; BIN, protein brassinosteroid insensitive; BZR1, Brassinazole resistant 1.

A total of 621 of the DEGs were annotated according to the KEGG database. Enrichment analysis of these genes revealed that most of genes in “energy metabolism” were down-regulated, while genes in “signal transduction” and “folding, sorting, and degradation” were up-regulated. The number of up-regulated genes in “carbohydrate metabolism” was slightly higher than the number of down-regulated genes (Figure 5). In the “environmental information processing” category, “MAPK signaling pathway,” “calcium signaling pathway,” and “plant hormone signal transduction” were active in response to drought, among them, fifteen DEGs were highly similar to MAPK genes, including MAP2K1, MAPK1-2, and MAPK1-3, and all the genes encoding MAPKs were up-regulated during the drought treatment. In the plant hormone signal transduction pathway, we found that protein phosphatase 2C (PP2C) (contig38492, contig19734, contig01077), serine/threonine protein kinase SRK2 (SnRK2) (contig40493, contig14094) and ABA responsive element binding factor (ABF) (contig17149, L1-GH8N3EB01DW119) of the ABA signal pathway, ethylene insensitive protein (EIN3) (contig21118) of the ethylene pathway, jasmonate ZIM domain-containing protein (JAZ) (contig39383) of the JA signal pathway, and auxin-responsive protein (IAA) (contig42235, contig38587, contig18621), BSK (contig41577) of BR signal pathway were affected significantly (fold change >4). Based on the pathway analysis, the hypothetical model of coordinative signaling pathway networks on drought response in sheepgrass were proposed (Figure 6). More information from the pathway analysis is reported in Table S7.

### KOG categories

**Figure 7:**
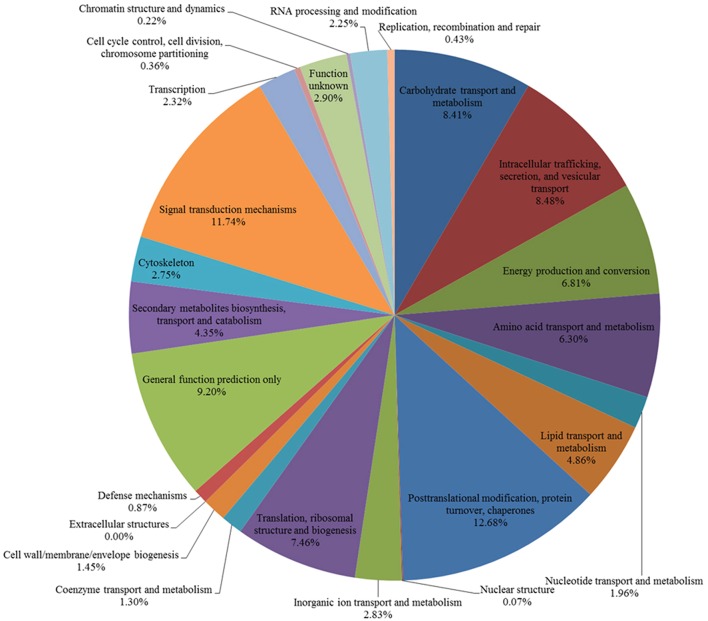
**KOG functional classification**. The 7320 DEGs were aligned with the KOG database to predict their possible functions. A total of 1380 genes were assigned to 24 categories.

The DEGs were further annotated based on the EuKaryotic Orthologous Groups (KOG) database. Of these DEGs, 1380 contigs were assigned functional annotations grouped into 24 categories. A total of 12.68% of 1380 contigs were involved in “Posttranslational modification, protein turnover, chaperones,” 11.74% in “Signal transduction mechanisms,” 6.81% in “Energy production and conversion” and 8.41% in “Carbohydrate transport and metabolism” (Figure 7). “Serine/threonine protein kinase,” “Serine/threonine protein phosphatase” and “Mitogen-activated protein kinase” were the top three categories in “Signal transduction mechanisms” (Table S8). However, we found many contigs that could not be annotated according to the NCBI, GO, KEGG, and KOG databases, indicating that a large number of sheepgrass-specific genes exist.

### Transcription factor response to drought

**Figure 8:**
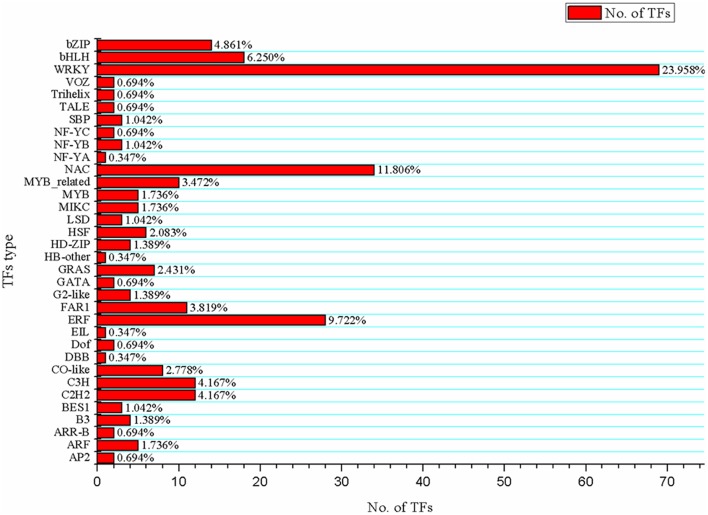
**Identification of transcription factors from 7320 DEGs**. Abscissa indicates the number of genes assigned to a specific family; ordinate indicates the transcription factor types; the rate of each type is shown on the right of the bar.

**Figure 9:**
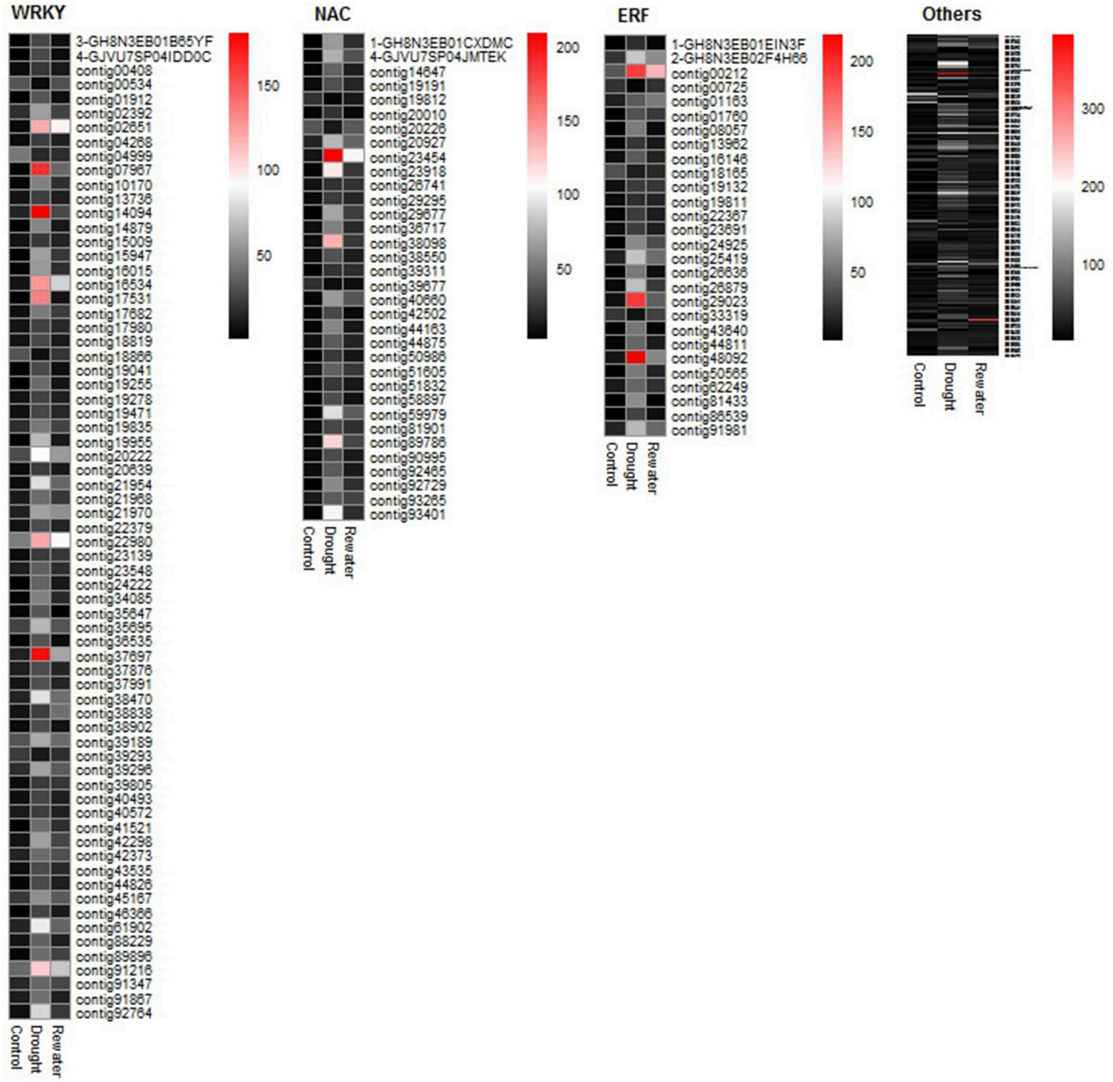
**Heatmap of transcription factor expression abundance**. The RPKM value of each gene is used to plot the heatmap.

TFs are implicated in diverse biological functions such as growth, development and stress tolerance. Their ability to control the expression of numerous genes has aroused much scientific interest. A total of 288 TFs grouped into 34 families were identified among the DEGs. The top three TF families were WRKY (23.958%), NAC (11.806%), and ERF (9.722%), followed by bHLH (6.250%), bZIP (4.861%), and others (Figure 8). In combination with GO analysis, we found that these TFs were involved in several biological processes, including plant hormone signal transduction and stress responses. Most of these TFs were induced by drought and returned to lower levels after rewatering (Figure 9, Table S9).

### Validation of RNA-seq data by qRT-PCR

**Figure 10:**
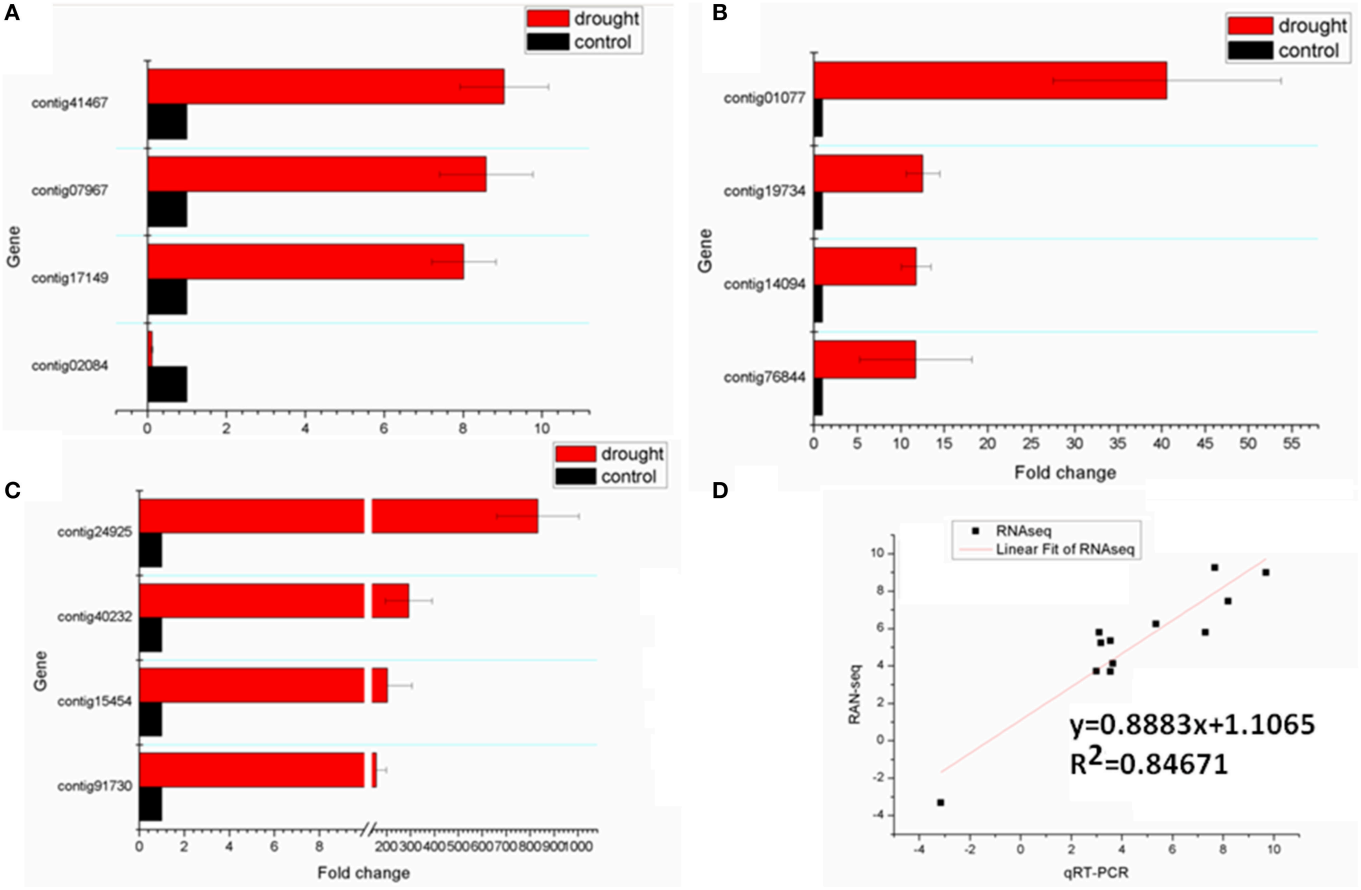
**Validation of RNA-seq results by qRT-PCR**. **(A–C)** The expression levels of 12 selected genes in sheepgrass seedlings under drought and control. **(D)** Correlation analysis indicating the relationship between qRT-PCR results (log2 fold-change; X-axis) of the expression abundance of 12 selected genes and the corresponding data from RNA-seq analysis (Y-axis).

To confirm the results from RNA-seq, we selected 12 contigs for qRT-PCR analysis (primers see Table S10). Scatterplots were generated using the log2 fold-change of RNA-seq and qRT-PCR results. The correlation of the two analyses was evaluated by the Linear Fit test. The results from the qRT-PCR closely matched the results of RNA-seq (*R*^2^ = 0.8467), which supported the reliability of our RNA-seq data (Figure 10, Additional images 1, 2).

## Discussion

Sheepgrass, an important forage grass, adapts well to drought, cold, saline and alkaline conditions. For better understanding of the molecular mechanism of its adaptability to diverse adverse environments, studies on saline stress, saline-alkali stress, and cold stress have been completed with the development of microarray chips and high-throughput sequencing (Jin et al., 2008; Chen et al., 2013; Sun et al., 2013; Zhai et al., 2014). However, little information is available about drought stress. Here, we focused on the effects of different moisture levels on the plant response. In this study, we performed a comparative analysis of the gene expression changes of sheepgrass under control, drought stress, and rewatering conditions. The results reported in this research provide a comprehensive overview of gene expression changes in sheepgrass in response to water changes and a platform for functional gene selection in plant drought resistance.

### Stress responsive proteins play crucial roles in drought tolerance

Dehydrins, a group of proteins abundant in late embryogenesis proteins, accumulate in response to drought stress and low temperatures in plant tissues, especially in drought-tolerant plants (Close, 1997; Beck et al., 2007). Some reports have suggested that dehydrins assist the correct folding of both structural and functional proteins and inhibit lipid peroxidation (Hara et al., 2003; Liu and Jiang, 2010). A number of dehydrins were induced by progressive water deficit in tall fescue (*Schedonorus phoenix;* Jiang and Huang, 2002). Moreover, Guo et al. showed that dehydrin (*Dhn3)* was differentially expressed in two drought-tolerant barley genotypes under drought stress (Guo et al., 2009). The expression level of dehydrin (*Dhn3)* was highly increased when the leaf was slightly wilted in tolerant ryegrass or severely wilted in susceptible ryegrass (Liu and Jiang, 2010). This result indicates that the tolerant species respond to stress earlier than the sensitive species. Further, the accumulation of dehydrins improved the survival rate of plants under drought conditions (Borovskii et al., 2002; Porcel et al., 2005). The overexpression of wheat dehydrin (*Dhn5)* enhanced tolerance to osmotic stress in *Arabidopsis thaliana* (Brini et al., 2007). Here, we found eight putative dehydrins that were induced by drought in sheepgrass. In addition, two contigs encoding LEA were also up-regulated by drought stress. It is interesting that the expression of several cold-responsive proteins (e.g., cold shock protein CS66, cold acclimation protein, cold regulated protein, CBF II, COR39, COR413, and COR410) was apparently up-regulated under drought conditions (Table 2, Table S3). Furthermore, many putative sheepgrass-specific genes that were responsive to both drought stress and cold stress were revealed by comparative analysis with freezing-responsive genes (Chen et al., 2013; Table S13). This result indicates that these cold-responsive proteins play double roles in cold and drought stress, and there is widespread cross talk between the cold and drought stress response pathways in sheepgrass.

### Accumulation of compatible solutes

One tolerance mechanism is synthesizing compatible solutes when plants are in adverse environments. Osmolytes such as sugars, amino acids and amines, which accumulate under drought stress, maintain cell turgor, and stabilize protein structures. Trehalose is an osmolyte that accumulates in bacteria, fungi, and certain resurrection plants under abiotic stress (Seki et al., 2007). It has been demonstrated that trehalose can protect membranes and proteins from degradation to maintain the natural state of cells and organelles in response to various stresses (Crowe et al., 1998; Crowe, 2007; Delorge et al., 2014). The biosynthesis of trehalose includes two steps in plants, involving trehalose-6-phosphate synthase (TPS) and trehalose-6-phosphate phosphatase (TPP; Paul, 2007). In *Arabidopsis* thaliana, 11 TPS and 10 TPP genes were uncovered, with only *AtTPS1* encoding an active synthase (Leyman et al., 2001). The expression of *OsTPP1* and *OsTPP2* has been found to be induced by drought, ABA and chilling (Pramanik and Imai, 2005). Moreover, the overexpression of *AtTPS1* or *OsTPS1* enhanced the stress tolerance of transgenic lines (Avonce et al., 2004; Li et al., 2011). Proline, which acts as an osmoprotectant, stabilizer and scavenger, plays a multifunctional role in the defense mechanisms (Nanjo et al., 1999; Seki et al., 2007). Delta 1-pyrroline-5-carboxylate synthetase (P5CS) catalyzes the first two steps in proline biosynthesis (Hu et al., 1992). Transgenic lines overexpressing *OsP5CS* exhibited improved proline content and enhanced drought tolerance (Yamada et al., 2005b). In this study, a putative *TPP* gene (contig38471) and two P5CS encoding genes (contig60066, contig33109) were identified as exhibiting drought-inducible expression (Table 2).

### Scavenging of reactive oxygen species

Reactive oxygen species (ROS), formed by an electron transfer chain, are unstable molecules produced both as a result of normal aerobic metabolic processes and in response to abiotic stresses, including drought, high salinity, and low or high temperature stresses (Cruz de Carvalho, 2008). Under optimal growth conditions, the production of ROS remains at a low level, whereas the rate of production is dramatically elevated in response to stresses (Miller et al., 2010). Excessive ROS can lead to the irreversible oxidization of lipids, proteins, and nucleic acids (Li et al., 2012). Plants have evolved scavenging mechanisms to alleviate the destructive effects of ROS. ROS-scavenging enzymes, such as superoxide dismutase (SOD), peroxidase (POD), and catalase (CAT), play an important role in protecting plants against oxidative damage (Wang et al., 2005; Koussevitzky et al., 2008). Herein, we have revealed five POD-encoding genes that were observed to be up-regulated at least 32-fold by drought stress (Table 2). Moreover, several contigs of “response to oxidative stress” were induced at different levels (Table S6). These changes in expression are beneficial for improving the tolerance of Sheepgrass to drought stress.

### Plant hormone signal functions in plant stress tolerance

**Figure 11:**
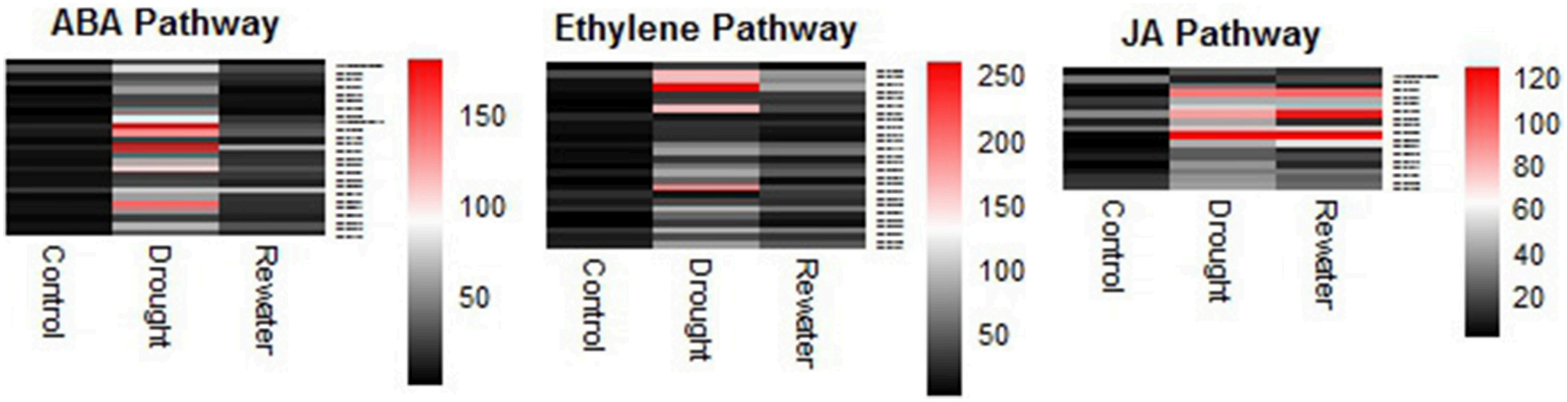
**Heatmap analysis of hormone-related genes under different conditions**. ABA means Abscisic acid; JA means jasmonic acid.

Many tolerance strategies are utilized by plants to resist or adapt to challenging environments. Plants transmit signals upon sensing soil water content changes to distant organs by both hydraulic signals and chemical signals (Wilkinson and Davies, 2010). Because of their importance in responding to the adverse effects of different stresses, plant hormone signals have attracted much attention. In this study, many genes of hormonal signaling pathways, including ABA, ethylene, JA, and other signaling pathways, were identified as being involved in the drought response in Sheepgrass (Figure 11, Table >S11).

ABA is essential for various stress responses, including stomatal closure, the expression of stress-responsive genes, and metabolic changes. The endogenous ABA level, which is determined by the rate of ABA biosynthesis and catabolism, fluctuates in response to environmental changes, especially drought and salt stresses (Seki et al., 2007). The enzyme 9-cis-epoxycarotenoid dioxygenase (NCED) plays a crucial role in the production of the precursor of ABA (Schwartz et al., 1997; Seki et al., 2007). In *Arabidopsis, AtNCED3* was found to be induced by drought stress, and the overexpression of *AtNCED3* enhanced plant drought tolerance (Iuchi et al., 2001). In addition, endogenous ABA levels are also regulated by deconjugation, conjugation and hydroxylation, which are catalyzed by β-glucosidase, glycosyl transferase and 8′-hydroxylase, respectively (Dietz et al., 2000; Saito et al., 2004; Lee et al., 2006; Jiang and Hartung, 2008; Okamoto et al., 2009; Xu Z. Y. et al., 2012; Danquah et al., 2014). In our research, two contigs encoding putative *NCEDs* were up-regulated more than 64-fold by drought (Table 2).

A dynamic balance of biosynthesis and degradation determines the amount of available cellular ABA (Danquah et al., 2014). In the absence of ABA, protein phosphatase 2Cs (PP2Cs) dephosphorylate and inactivate SNF1-related protein kinase 2s (SnRK2s), which are positive regulators of the ABA pathway. The PYR/PYL/RCAR-PP2C complex leads to the inhibition of PP2C activity after the binding of ABA to PYR/PYL/RCAR (ABA receptor), allowing the activation of SnRK2s that target TFs and ion channels and induce the transcription of ABA-responsive genes (Ma et al., 2009; Soon et al., 2012; Danquah et al., 2014). In our research, several components of the ABA pathway have been identified, such as PP2C, SnRK2, and ABF (Table S7). Moreover, many contigs of “ABA biosynthetic process” and “ABA mediated signaling pathway” were up-regulated by drought stress and returned to lower levels after rewatering (Table S11, Figure 11).

Elevated ethylene production can be induced by various types of stresses. In wheat leaves, after a loss of 9% of initial fresh weight, the production of ethylene increased more than 30-fold within 4 h (Apelbaum and Yang, 1981). The finding that plants overexpressing *OsETOL1* show reduced ethylene accumulation and reduced drought tolerance indicates that the ethylene level correlates positively with drought resistance (Du et al., 2014). Aminocyclopropane-1-carboxylic acid (ACC) synthase (ACS), which catalyzes S-adenosylmethionine (SAM) to ACC, the precursor of ethylene, is the rate-controlling enzyme in ethylene biosynthesis. MAPKs play important roles in the activation of ACS genes and ethylene signaling (Zhao and Guo, 2011; Kazan, 2015). ACC is converted to ethylene gas by ACC oxidase (ACO) in the subsequent step (Apelbaum and Yang, 1981; Wilkinson and Davies, 2010; Kazan, 2015). In this study, we found an ACC-encoding gene (contig15454) that was up-regulated more than 128-fold (Table 2).

Constitutive triple response 1 (CTR1), a Ser/Thr kinase, plays a critical role in ethylene signal transduction through its interaction with ethylene receptors. In the absence of ethylene, the receptors activate CTR1, which can inactivate the downstream components (EIN2 EIN3/EILs) by phosphorylation to negatively regulate the signals. CTR1 activity is repressed after the binding of ethylene to the receptors, which activates a transcriptional cascade response involving EIN2, EIN3, WRKY, and ethylene response factor (ERF) (Chao et al., 1997; Ju et al., 2012; Qiao et al., 2012; Shakeel et al., 2013; Kazan, 2015). In this research, we found that the genes of the “ethylene biosynthetic process,” “ethylene mediated signaling pathway,” and ERFs presented regular changes (Figure 11). More detailed information on these pathways is given in Table S11.

In addition, JAs have been implicated in modulating root hydraulic conductivity and promoting stomatal closure, thus contributing to drought resistance (Suhita et al., 2004; Munemasa et al., 2007; Sanchez-Romera et al., 2014). Here, several contigs of the “jasmonic acid biosynthetic process” were induced at different levels (Table S11).

It was reported previously that the crosstalk between BRs and ABA was existent in stress responses network (Choudhary et al., 2012). In this article, the expressions of BSKs and BINs in BR signal pathway were upregulated under drought stress in sheepgrass, these results indicate that the BRs play important role in complex regulated network.

### Why biomass decreases under drought stress

**Figure 12:**
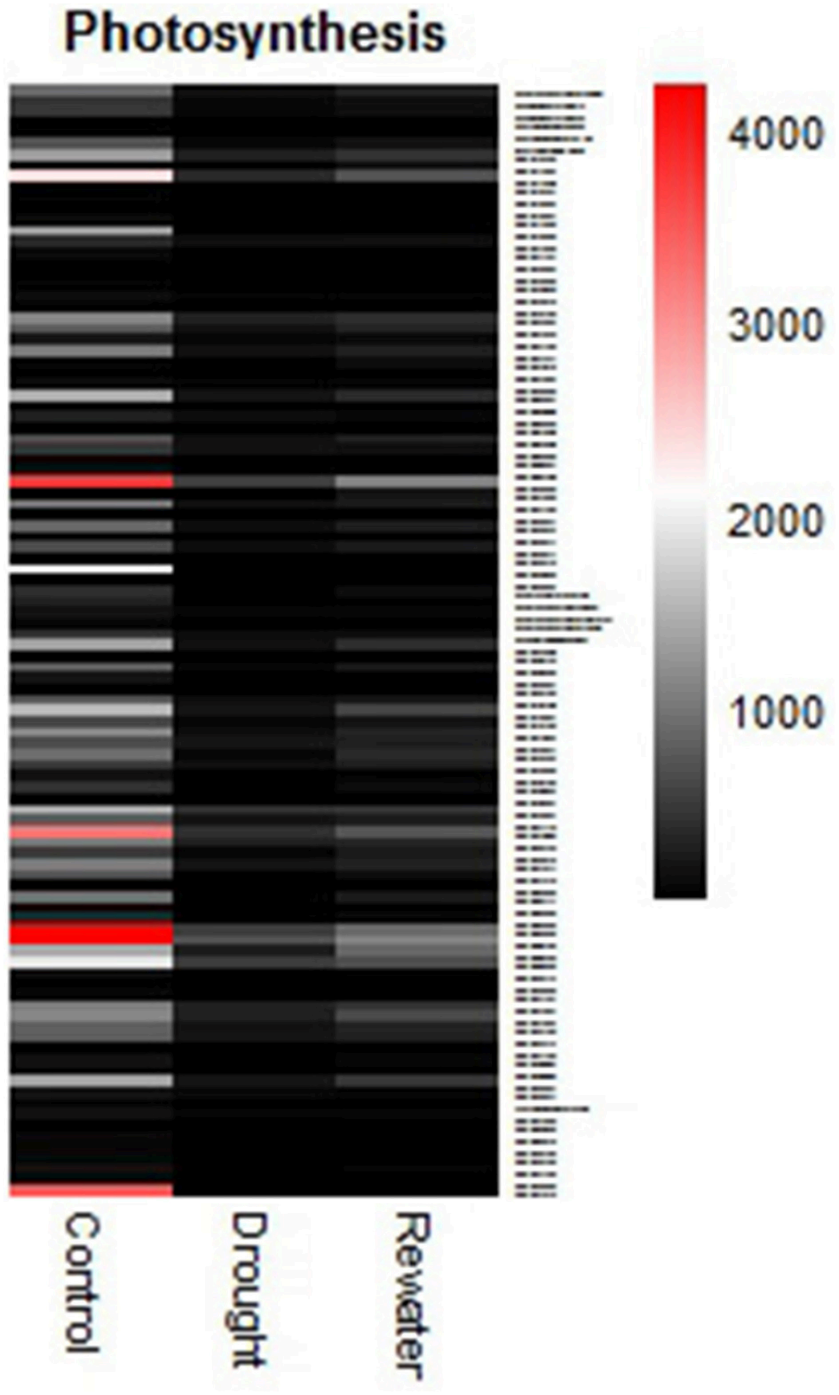
**The expression changes of photosynthesis-related genes**.

Our results indicated an extremely significant decrease in dry weight on the 28th day. Similar results were observed under saline-alkali (Na_2_CO_3_) and saline (NaCl) stresses and drought stress (red clover; Jin et al., 2008; Yates et al., 2014). The enzyme β-amylase catalyzes starch into soluble sugar, which can protect proteins and membranes. Daisuke Todaka et al. reported that water-limiting could enhance β-amylase activity in cucumber cotyledons (Todaka et al., 2000). Moreover, two members of the β-amylase family (BMY7 and BMY8), which have apparent transit peptides for chloroplast stromal localization, were induced by temperature shock. The induction of β-amylase expression was thought to be correlated with maltose accumulation (Kaplan and Guy, 2004). Nevertheless, the transcript abundance of rubisco small subunit was shown to decline rapidly from moderate to severe drought (Vu et al., 1999). In our research, most photosynthesis-related genes were down-regulated greatly, especially the rubisco large subunit (Figure 12, Table 2, Table S12). In contrast, the transcript abundances of β-amylase (4-GJVU7SP04ITTBI) and sugar transport protein (contig90018) were elevated (Table 2). These changes of gene expression are likely to be responsible for the biomass decline of the aboveground parts, although they were helpful in resistance to stress.

## Conclusion

The global investigation of the expression changes in sheepgrass under drought stress was carried out in this article. We revealed 680 putative sheepgrass-specific water responsive genes with potential application value for molecular breading. Interestingly, widespread cross talk is suggested between the cold and drought stress response pathways in sheepgrass, typical cold response CBF pathway is activated in drought stress, which indicated the pivotal role of key genes in various signal pathways. In addition, we found that many potential targets, such as dehydrin protein and late embryogenesis abundant protein (LEA), played crucial roles in drought response. Functional annotation and classification analysis showed that many stress-related genes and plant hormone signaling pathway genes responded strongly to drought stress. Our research will provide new insight into the molecular mechanism of the sheepgrass response to drought stress. Moreover, the data from our study provide a valuable gene resource for future work.

## Author contributions

PL, TM, LC and GY participated in the design of the study and performed the molecular biology experiments of RNA-seq. PZ have made substantial contributions to analysis of RNA-seq data. PZ, JJ, XL and DQ participated in the design of the non-sequencing experiments and performed the statistical analysis. SC, GL and LC conceived of the study, and helped to draft the manuscript. All authors read and approved the final manuscript.

### Conflict of interest statement

The authors declare that the research was conducted in the absence of any commercial or financial relationships that could be construed as a potential conflict of interest.

## Supplementary material

Table S1 **Soil water content and plant morphological data**.

Table S2 **The results of mapping**.

Table S3 **A list of differentially expressed genes between drought stress and control**.

Table S4 **A list of differentially expressed genes between rewatering and drought stress**.

Table S5 **A list of differentially expressed genes between rewatering and control**.

Table S6 **GO analysis of differentially expressed genes**.

Table S7 **KEGG pathway analysis of differentially expressed genes**.

Table S8 **KOG classification of differentially expressed genes**.

Table S9 **A list of putative transcription factors**.

Table S10 **Primers for qRT-PCR**.

Table S11 **A list of phytohormone-related genes and corresponding transcript abundance**.

Table S12 **A list of photosynthesis-related genes and corresponding transcript abundance**.

Table S13 **Comparative analysis of drought-responsive genes with freezing-responsive genes**.

Table S14 **Identification of sheepgrass-specific water responsive genes**.

Additional image 1 **Depth of sequence (A) Control; (B) Drought stress; (C) Rewater**.

Additional image 2 **Statistic of original reads**. L1: Control; L2: Drought stress; L3: Rewater.
