# Supplementary material for: New Insights on Drought Stress Response by Global Investigation of Gene Expression Changes in Sheepgrass (Leymus chinensis)
Source: Front Plant Sci. 2016 Jun 30;7:954. doi: 10.3389/fpls.2016.00954 (PMC4928129; doi:10.3389/fpls.2016.00954)
Supplement: Table S2 — The results of mapping. [file Table2.DOCX]

Table S2 The results of mapping

| Map to gene | | | | | L1 | | | |  | L2 | | | | |  | | L3 | |  | |  |
| --- | --- | --- | --- | --- | --- | --- | --- | --- | --- | --- | --- | --- | --- | --- | --- | --- | --- | --- | --- | --- | --- |
|  | Reads number | | | | | percentage | | Reads number | | | | percentage | | Reads number | | | | Reads number | | | |
| clean reads | | | 6180085 | 100% | | | 6177255 | | | | 100% | | 5483463 | | | 100% | | | |  |  |
| Total mapped reads | | | 5360719 | 86.74% | | | 5577575 | | | | 90.29% | | 4959985 | | | 90.45% | | | |  |  |
| Total unmapped reads | | | 819366 | 13.26% | | | 599680 | | | | 9.71% | | 523478 | | | 9.55% | | | |  |  |
| Unique_match | | | 1981883 | 32.07% | | | 2024294 | | | | 32.77% | | 1795132 | | | 32.74% | | | |  |  |
| Mutliple_match | | | 3378836 | 54.67% | | | 3553281 | | | | 57.52% | | 3164853 | | | 57.72% | | | |  |  |
| Perfect match | | | 3310916 | 53.57% | | | 3167897 | | | | 51.28% | | 2970286 | | | 54.17% | | | |  |  |
| <=5bp mismatch | | 1982565 | | 32.08% | | | 2334047 | | | | 37.78% | | 1927450 | | | 35.15% | | | |  |  |

Note: L1(leaf control), L2(leaf drought), L3(leaf rewater)
