# Supplementary figures and images for: New Insights on Drought Stress Response by Global Investigation of Gene Expression Changes in Sheepgrass (Leymus chinensis)
Source: Front Plant Sci. 2016 Jun 30;7:954. doi: 10.3389/fpls.2016.00954 (PMC4928129; doi:10.3389/fpls.2016.00954)

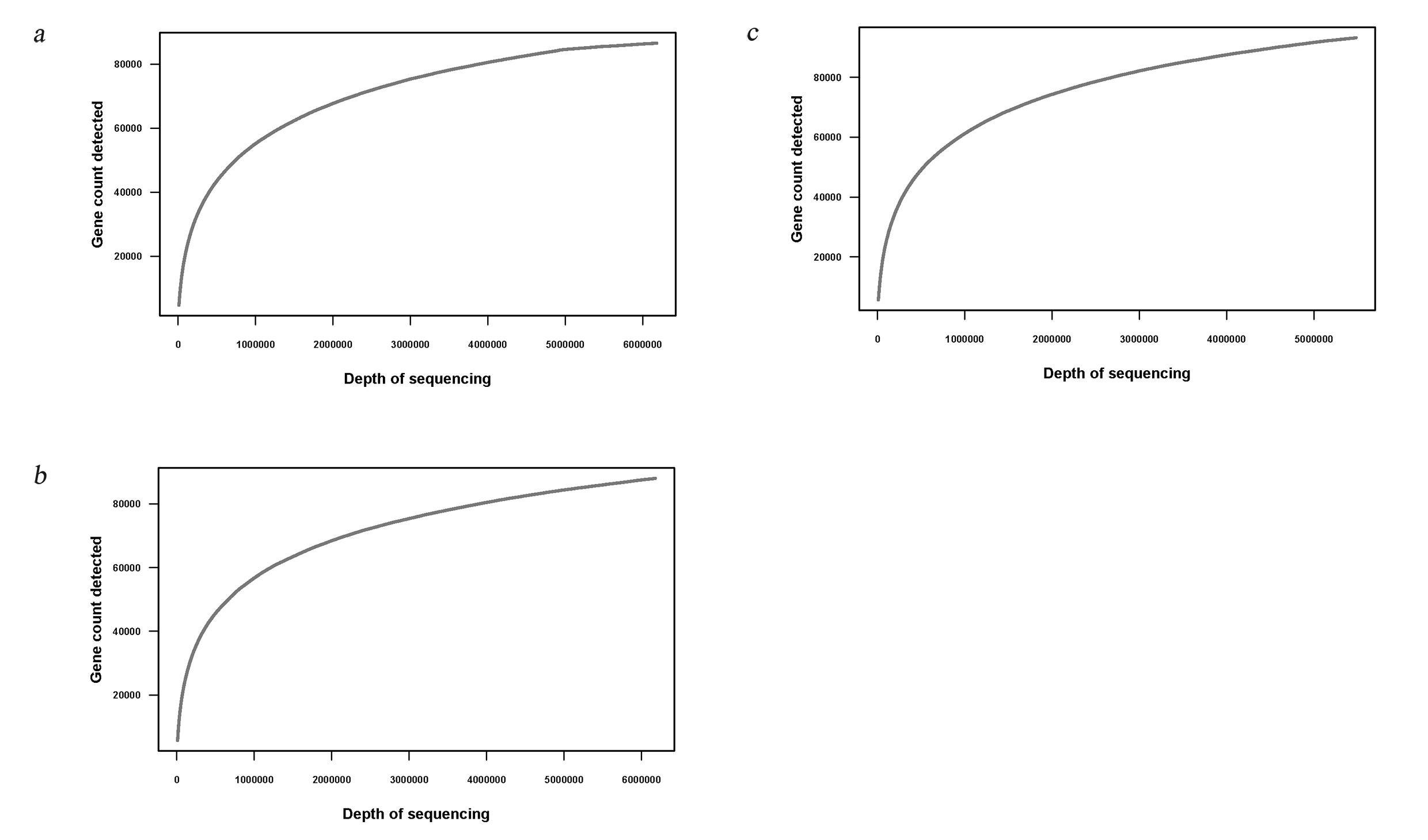

Supplement: Additional image 1 — Depth of sequence (A) Control; (B) Drought stress; (C) Rewater. [file Image1.TIF]

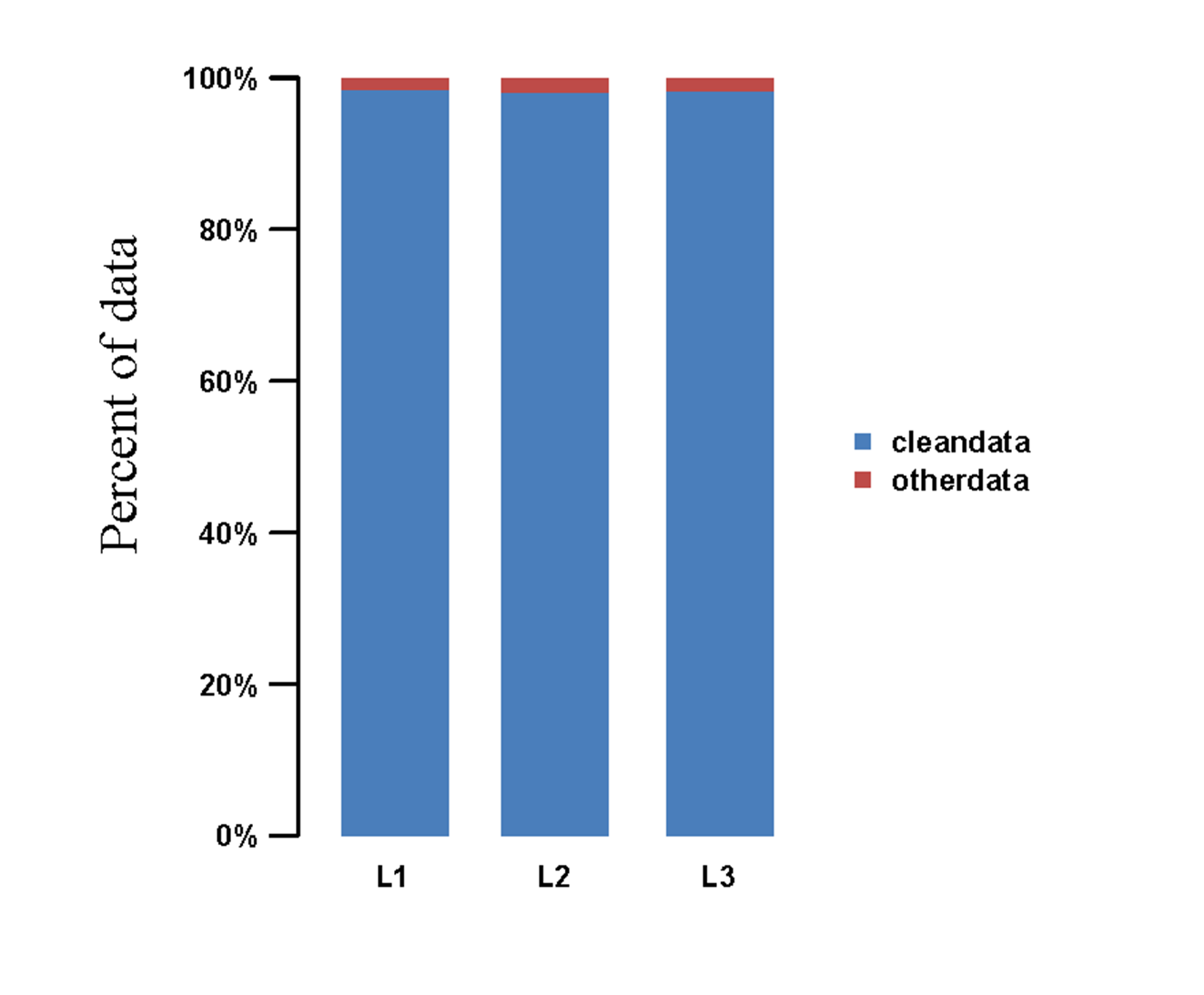

Supplement: Additional image 2 — Statistic of original reads. L1: Control; L2: Drought stress; L3: Rewater. [file Image2.TIF]
